# Long-term effects of abatacept on atherosclerosis and arthritis in older vs. younger patients with rheumatoid arthritis: 3-year results of a prospective, multicenter, observational study

**DOI:** 10.1186/s13075-024-03323-8

**Published:** 2024-04-17

**Authors:** Zento Yamada, Sei Muraoka, Mai Kawazoe, Wataru Hirose, Hajime Kono, Shinsuke Yasuda, Takahiko Sugihara, Toshihiro Nanki

**Affiliations:** 1 Division of Rheumatology, Department of Internal Medicine, Toho University School of Medicine, 6-11-1 Omori-nishi, Ota-ku, Tokyo, 143-8541 Japan; 2 Hirose Clinic of Rheumatology, 2-14-7 Midoricho, Tokorozawa, 359-1111 Saitama Japan; 3 Department of Internal Medicine, Teikyo University School of Medicine, 2-11-1, Kaga, Itabshi- ku, Tokyo, 173-8606 Japan; 4 Department of Rheumatology, Endocrinology and Nephrology, Graduate School of Medicine, Faculty of Medicine, Hokkaido University, Kita 14, Nishi 5, Kita-ku, Sapporo, 060-8648 Japan; 5 Department of Medicine and Rheumatology, Tokyo Metropolitan Geriatric Hospital, 35-2, Sakaecho, Itabashi-ku, Tokyo, 173-0015 Japan

**Keywords:** Abatacept, Rheumatoid arthritis, Atherosclerosis, Elderly, Retention rate

## Abstract

**Background:**

We aimed to reveal the effect of abatacept (ABT) on atherosclerosis in rheumatoid arthritis (RA) patients, 3-year efficacy for arthritis, and safety in a population of older vs. younger patients.

**Methods:**

In this open-label, prospective, observational study, patients were stratified into four groups: younger (20–64 years old) and older (≥ 65 years) patients taking ABT (AY and AO) and conventional synthetic disease-modifying antirheumatic drugs (csDMARDs) (CY and CO). Primary endpoints were change from baseline in mean intima-media thickness (IMT) of the common carotid artery, IMT max (bulbus, bifurcation, and internal and common carotid artery), and plaque score at Week 156. Disease activity, retention rate, and adverse effects were also evaluated.

**Results:**

The ABT group (AY + AO) tended to have smaller increases in mean IMT, max IMT, and plaque score than the csDMARD group (CY + CO) at Week 156, although the differences between groups were not statistically significant. Multivariate analysis showed significantly lower increases in plaque score with ABT than with csDMARDs, only when considering disease activity at 156 weeks (*p* = 0.0303). Proportions of patients with good or good/moderate European League Against Rheumatism response were higher in the ABT group, without significant difference between older and younger patients. No significant differences were observed in ABT retention rates between older and younger patients. Serious adverse effects, especially infection, tended to be more frequent with ABT than with csDMARDs, although no significant differences were found.

**Conclusions:**

ABT may decelerate atherosclerosis progression and may be useful for patients with high risk of cardiovascular disease, such as older patients.

**Trial registration number:**

UMIN000014913.

**Supplementary Information:**

The online version contains supplementary material available at 10.1186/s13075-024-03323-8.

## Background

Rheumatoid arthritis (RA) is a chronic, progressive, possibly debilitating, autoimmune disease without cure [1]. The main features of RA are higher frequency among women than men (ratio of 3 to 1), systemic involvement, joint inflammation and destruction, predominance in small joints, deformity, loss of function, and disability [1]. The global incidence of RA is estimated at 1% [2] and that in Japan at 0.75% [3]. A recent epidemiological study in Japan reported that the main population affected by RA consisted of elderly patients in their 60s and that the prevalence continued to increase with age until the 80s [3]. Despite the higher prevalence of RA in older age [3], there is a paucity of prospective studies evaluating treatment outcomes among the elderly [4].

Developing and approving novel therapies, i.e., biologic and targeted synthetic disease-modifying antirheumatic drugs (b/tsDMARDs), has remarkably improved patient prognosis and quality of life [5]. Elderly patients with RA are at a higher risk of developing infections due to treatment with bDMARDs [6, 7] and Janus kinase inhibitors may increase the incidence of malignancy [8]. Furthermore, elderly patients tend to present comorbidities and receive multiple medications. These factors may hinder treatment and make it difficult to control disease activity. Nevertheless, data remain inconclusive regarding the risk of infection, malignancy, or other treatment-related complications associated with specific b/tsDMARDs [9, 10].

Abatacept (ABT), an inhibitor of T-cell activation by blocking cluster of differentiation (CD)80/86-CD28 interaction, is a widely used biologic agent for the treatment of RA [11]. A recent real-world data study using multiple databases showed that among patients with a mean age of 52.6 years, the risk of specific cancers and infections with ABT did not differ significantly from that with other b/tsDMARDs [12]. In an all-cases post-marketing surveillance in Japanese patients treated with ABT, the incidence of overall adverse reactions and infections, and serious adverse reactions was significantly lower in non-elderly patients than in elderly patients. However, the incidence of serious infections was not significantly different between non-elderly and elderly patients [13].

Patients with RA are at an increased risk of cardiovascular disease secondary to atherosclerosis [14, 15], particularly among patients with poorly controlled RA, due to chronic inflammation [16]. Cardiovascular mortality accounts for roughly 50% of the deaths among patients with RA [17, 18]. Theoretically, agents that inhibit inflammation, such as b/tsDMARDs, should also reduce cardiovascular risk [19]. Indeed, it has been reported that tumor necrosis factor (TNF) inhibitors reduce the incidence of cardiovascular events in RA patients [20]. However, evidence is limited, particularly for long-term use. Specifically, the long-term effect of ABT on arterial stiffness, and thus cardiovascular risk, has not been reported. Data are also conflicting because it has been reported that some DMARDs may indirectly or directly contribute to atherosclerosis [19].

In contrast to other agents, such as TNF and interleukin-6 inhibitors, directly inhibiting the inflammatory mediators, ABT acts as a T-cell inhibitor, more upstream of the inflammatory cascade. It was reported that T-cell CD80/86-CD28 co-stimulation is key to the onset of accelerated atherosclerosis in mice [21]. Given its point of action, ABT may have an anti-atherosclerotic and survival-improving effect aside from the effect on joint swelling and inflammation [22].

RA is a chronic disease that requires long-term treatment, and there seems to be a large variability across different drugs. Thus, it is vital to attain disease control by considering each patient’s needs and selecting the medication that would lead to the most benefits in terms of joint and cardiovascular outcomes. We conducted a prospective observational study (Investigation of the Effects of Abatacept on Rheumatoid Arthritis: Analysis of Efficacy on Arthritis and Atherosclerosis [ABT-ATS study]) to evaluate the efficacy and safety of ABT in older vs. younger patients after 24 weeks of treatment and found that ABT was both efficacious and safe in both older vs. younger patients with RA who were refractory to conventional synthetic disease-modifying antirheumatic drugs (csDMARDs) [23].

In the present study, we assessed the efficacy of ABT on atherosclerosis. Additionally, we compared the ABT retention rate and adverse events (AEs) in older vs. younger patients and identified cardiovascular risk factors, while continuing to evaluate the efficacy in terms of disease activity and safety of long-term treatment with ABT for up to 3 years (156 weeks) in older vs. younger patients with RA.

## Methods

### Study design

The study design and methods have been previously described [23]. This study was an open-label, prospective, observational study conducted at 31 centers across Japan (see the appendix in Additional file 1). The study protocol was approved by the Ethics committee of Faculty of Medicine, Toho University (Approval number: A20114_A17112_A16017_27038), Teikyo University (14–061) and ethics committees of collaborative institutions and registered at UMIN Clinical Trial Registry under the identifier UMIN000014913. The ethical principles of the Declaration of Helsinki and ethical guidelines for clinical research of the Ministry of Health, Labour and Welfare, Japan guided the study conduct. All participants provided informed consent for study participation at the time of enrollment.

### Patients

Included patients were men and women, aged 20 years or older, with RA diagnosed according to the 2010 American College of Rheumatology/European League Against Rheumatism (EULAR) classification criteria [24], who had a history of being refractory to treatment with csDMARDs, were naïve to treatment with bDMARDs, and who provided written informed consent. Excluded patients were those with malignant tumors, active infections, pregnancy, or those deemed ineligible for study participation by the physician.

### Interventions and treatment

This is a non-interventional study, and treatment was appointed by the treating physician at their discretion. Patients could either initiate ABT (ABT group), or those in the csDMARD group could add a csDMARD to their prescribed csDMARD baseline regimen, or switch to a new csDMARD.

Patients were stratified by age and treatment into four groups: younger (20 to 64 years old) and older (65 years or older) patients taking ABT (AY and AO), as well as younger and older patients taking csDMARD (CY and CO). ABT was administered by intravenously or subcutaneously. Intravenous ABT was administered at the recommended dose [25] at the start of treatment (baseline), 2 and 4 weeks after baseline, and then at 4-week intervals for the study duration. The recommended dose was administered in 100 ml of 0.9% sodium chloride aqueous solution and was based on patient body weight as follows: 500 mg for patients weighing < 60 kg, 750 mg for patients weighing ≥ 60 kg and ≤ 100 kg, and 1000 mg for patients weighing > 100 kg. For subcutaneous ABT, loading dose of intravenous ABT could be administrated on the first day, followed by 125 mg subcutaneous ABT, and then at a dosage of 125 mg weekly, or it could be administered subcutaneous ABT alone at the physician’s discretion. csDMARDs, glucocorticoids, and non-steroidal anti-inflammatory drugs could be added or changed, or their dosages modified based on the treating physician’s discretion [23]. Patients who discontinued ABT treatment or those taking csDMARDs who initiated bDAMRDs or Janus kinase inhibitors were withdrawn from the study.

### Study endpoints

The primary endpoints were change from baseline in mean intima-media thickness (IMT) of the common carotid artery, IMT max values of the bulbus, bifurcation, internal and common carotid artery, and plaque score [26], measured by carotid duplex ultrasound, compared between the ABT and csDMARD groups and in younger and older patients at Week 156.

Secondary endpoints were the following: change from baseline in mean IMT of the common carotid artery, IMT max values of the bulbus, bifurcation, internal and common carotid artery, and plaque score, measured by carotid duplex ultrasound, compared between the ABT and csDMARD groups and in younger and older patients at Weeks 52 and 104; change from baseline in pulse wave velocity (PWV) compared between the ABT and csDMARD groups and in younger and older patients at Weeks 52, 104, and 156; good and good or moderate response according to the EULAR response criteria at Weeks 52, 104, and 156; changes from baseline (Weeks 52, 104, and 156) in disease activity score as measured across 28 joints using the erythrocyte sedimentation rate (DAS28-ESR), Simple Disease Activity Index (SDAI), and Clinical Disease Activity Index (CDAI). Changes in the Health assessment questionnaire (HAQ) at Weeks 52, 104, and 156; and ABT retention rate at Weeks 52, 104, and 156. Safety was evaluated based on the incidence of AEs and serious AEs (SAEs). SAEs were defined as a fatal event, a life-threatening event, an event leading to hospitalization, an event leading to permanent or significant disability, or any other important medical event.

### Data collection

Data were collected in case registration forms. Baseline data on demographic and clinical characteristics (duration of RA, RA treatment history, rheumatoid factor, and anti-cyclic citrullinated peptide antibody); carotid duplex ultrasound and PWV at baseline, Weeks 52, 104, and 156; and efficacy and laboratory parameters at baseline and Weeks 52, 104, and 156 were collected.

### Statistical analysis

As measures to avoid bias, we used a multivariate analysis when analyzing atherosclerosis, and for disease activity we used propensity-score matching analysis. Analyses were conducted on the per-protocol population. Data are expressed as mean ± standard deviation. All statistical analyses were performed using R software, version 3.6.2 (R Foundation for Statistical Computing, Vienna, Austria). Comparisons were performed using Student’s t-test for continuous variables and the chi-squared test for categorical variables. In all analyses, *p* < 0.05 was considered statistically significant.

A multivariate analysis was conducted with a multivariate linear regression model to assess the factors influencing the extent of changes in plaque score, considering the effects of sex, age, body mass index, smoking, diabetes mellitus, hypertension, dyslipidemia, disease activity of RA, and ABT use. Propensity-score matching using baseline characteristics (including tender joint counts, swollen joint counts, C-reactive protein, ESR, physician’s visual analogue scale, patient’s visual analogue scale, proportion of methotrexate use, proportion of glucocorticoid use, disease duration, rheumatoid factor, and anti-cyclic citrullinated peptide antibody) was also applied for this analysis. Missing data led to pairwise deletion. To compare the ABT retention rate between younger and older patients, the log-rank test was used, and the results are shown using a Kaplan–Meier curve.

## Results

### Patient disposition and characteristics

Of the 219 patients screened at enrollment, three dropped out, and 216 patients were classified into four groups by age and treatment (AY, *n* = 52; AO, *n* = 73; CY, *n* = 41; CO, *n* = 50). At 156 weeks, patients who completed the study in the AY group were 25; in the AO group, 32; in the CY group, 32; and in the CO group, 29 (see Additional file 2, Supplemental Fig. 1).

Table 1 summarizes patient background characteristics at baseline in each group. In the AY and AO groups, patients had a mean ± SD age of 51.9 ± 11.2 and 75.0 ± 5.8 years, and in the CY and CO groups, 52.1 ± 9.3 and 74.1 ± 6.2 years. RA disease activity ratings (DAS28-ESR, SDAI, CDAI), and physical function ratings (HAQ) were higher in both younger and older ABT-treated patients. Atherosclerosis parameters (mean IMT, max IMT, plaque score, PWV) were higher in the older patients in both the ABT and csDMARD groups.

**Table 1:** Baseline characteristics of patients with rheumatoid arthritis, according to age and treatment group

	Abatacept		csDMARDs				Total *n* = 216	
	AY; *n* = 52	AO; *n* = 73	CY; *n* = 41		CO; *n* = 50			
Age (years)	51.9 ± 11.2	75.0 ± 5.8^‡^	52.1 ± 9.3		74.1 ± 6.2^$^		64.9 ± 13.8	
Female sex	42 (80.7)	59 (80.8)	36 (87.8)		43 (86.0)		180 (83.3)	
DAS28-ESR	4.5 ± 1.1	5.2 ± 1.2^‡^	3.3 ± 1.0^*^		3.7 ± 1.0^†^		4.3 ± 1.3	
SDAI	20.9 ± 9.7	25.1 ± 11.8	12.0 ± 6.0^*^		13.7 ± 7.4^†^		19.0 ± 10.8	
CDAI	19.4 ± 8.6	22.7 ± 10.8	11.7 ± 5.9^*^		13.1 ± 7.1^†^		17.6 ± 9.8	
HAQ score	0.6 ± 0.6	1.0 ± 0.8^‡^	0.3 ± 0.4^*^		0.6 ± 0.8^†^		0.7 ± 0.8	
RF-positive	44 (84.6)	63 (86.3)	31 (75.6)		36 (72.0)^†^		174 (80.6)	
ACPA-positive	47 (90.4)	62 (84.9)	32 (78.1)		37 (74.0)		178 (82.4)	
Steroid use	19 (36.5)	39 (53.4)	10 (24.4)		18 (36.0)		86 (39.8)	
Prednisolone daily dose (mg)	5.1 ± 3.5	6.2 ± 4.7	3.7 ± 1.6		3.9 ± 1.4^†^		5.2 ± 3.8	
MTX use	41 (78.8)	37 (50.7)	28 (68.3)		23 (46.0)		129 (59.7)	
Additional treatment initiated during this study								
Abatacept	52 (100)	73 (100)						
MTX	-	-	10 (24.4)		10 (20.0)		-	
Salazosulfapyridine	-	-	2 (4.9)		7 (14.0)		-	
Bucillamine	-	-	11 (26.8)		12 (24.0)		-	
Tacrolimus	-	-	2 (4.9)		5 (10.0)		-	
Iguratimod	-	-	15 (36.6)		14 (28.0)		-	
Leflunomide	-	-	1 (2.4)		2 (4.0)		-	
Mean intima-media thickness of common carotid artery (mm)								
Left	0.689 ± 0.214	0.798 ± 0.181^‡^	0.642 ± 0.118		0.803 ± 0.145^$^		0.743 ± 0.183	
Right	0.656 ± 0.198	0.798 ± 0.252^‡^	0.630 ± 0.098		0.786 ± 0.144^$^		0.728 ± 0.205	
Max intima-media thickness of common carotid artery, bulbus, bifurcation, and internal carotid artery (mm)								
Left	0.866 ± 0.293	0.974 ± 0.253^‡^	0.802 ± 0.162		1.012 ± 0.246^$^		0.923 ± 0.258	
Right	0.811 ± 0.274	1.001 ± 0.435^‡^	0.791 ± 0.121		0.991 ± 0.239^$^		0.912 ± 0.323	
Plaque score	2.062 ± 1.961	4.488 ± 2.743^‡^	1.803 ± 1.735		4.305 ± 2.471^$^		3.346 ± 2.615	
Pulse wave velocity (cm/s)								
Left	1269.4 ± 212.9	1777.6 ± 440.0^‡^	1311.1 ± 190.9		1666.2 ± 381.4^$^		1540.5 ± 406.4	
Right	1283.1 ± 233.4	1738.7 ± 403.5^‡^	1325.0 ± 194.4		1660.0 ± 372.6^$^		1531.0 ± 382.9	

Results are shown as mean ± standard deviation or n (%). *p* < 0.05, ^*^AY vs. CY, ^†^AO vs. CO, ^‡^AY vs. AO, ^$^CY vs. CO. Abbreviations: ACPA: anti-cyclic citrullinated peptide antibody; AO: older patients taking abatacept; AY: younger patients taking abatacept; CDAI: clinical disease activity index; CO: older patients taking conventional synthetic disease-modifying antirheumatic drugs; csDMARDs: conventional synthetic disease-modifying antirheumatic drugs; CY: younger patients taking conventional synthetic disease-modifying antirheumatic drugs; DAS28: disease activity score in 28 joints; ESR: erythrocyte sedimentation rate; HAQ: Health Assessment Questionnaire; MTX: methotrexate; SDAI: simple disease activity index

Supplemental Table 1 (see Additional file 2) shows baseline data for ABT and csDMARD groups, young and old combined. Patients in the ABT group had significantly higher baseline DAS28-ESR, SDAI, CDAI, and HAQ, as well as greater proportions of patients positive for rheumatoid factor and use of steroids than those in the csDMARD group.

### Primary endpoints

#### Efficacy of ABT on atherosclerosis

Changes in mean IMT of the common carotid artery, max IMT of the common carotid artery, bulbus, bifurcation, and internal carotid artery, plaque score, and PWV at Week 156 in ABT group (AY + AO) and csDMARD group (CY + CO) are shown in Table 2. The ABT group tended to have smaller increases in mean IMT, max IMT, and plaque score than the csDMARD group, although the differences between groups were not statistically significant. Comparison of AY vs. CY and AO vs. CO at Weeks 52, 104 and 156, and AY + AO vs. CY + CO at Weeks 52 and 104 are shown in Additional file 2, Supplemental Table 2. At Week 156, plaque score was significantly more increased with CO compared with AO (*p* = 0.0302). Overall, there was no consistent trend in PWV.

**Table 2:** Intima-media thickness, plaque score, and pulse wave velocity changes at Week 156

	Abatacept (AY + AO)	csDMARDs (CY + CO)	*P* value
Mean intima-media thickness of common carotid artery			
Left	0.022 ± 0.115	0.031 ± 0.116	0.6525
Right	0.019 ± 0.125	0.034 ± 0.126	0.5163
Max intima-media thickness of common carotid artery, bulbus, bifurcation, and internal carotid artery			
Left	0.008 ± 0.149	0.020 ± 0.215	0.7021
Right	0.003 ± 0.208	0.018 ± 0.203	0.6817
Plaque score	-0.446 ± 1.662	0.227 ± 1.915	0.0544
Pulse wave velocity			
Left	38.9 ± 384.5	73.0 ± 357.5	0.6092
Right	60.1 ± 242.1	32.3 ± 196.8	0.4868

Results are reported as change of the measured value compared with the baseline value. Abbreviations: AO: older patients taking abatacept; AY: younger patients taking abatacept; CO: older patients taking conventional synthetic disease-modifying antirheumatic drugs; csDMARDs: conventional synthetic disease-modifying antirheumatic drugs; CY: younger patients taking conventional synthetic disease-modifying antirheumatic drugs

#### Atherosclerosis and cardiovascular risk factors

Because treatment in the ABT group showed smaller increases of plaque score at Week 156, we performed multivariate analysis, considering sex, age, body mass index, smoking, diabetes mellitus, hypertension, dyslipidemia, and the disease activity of RA, which are known to have an effect on atherosclerosis. Results of multivariate analysis of plaque score at Week 156, considering the effects of sex, age, body mass index, smoking, diabetes mellitus, hypertension, dyslipidemia, disease activity by DAS-28-ESR at baseline, and ABT use, showed that women had a significantly lower increase of plaque score (*p* = 0.0264) than men. Hypertension was a significant risk factor for increased plaque score (*p* = 0.0230). Administration of ABT did not show a statistically significant difference, but the changes in plaque score were small (*p* = 0.0632). Next, we applied DAS-28-ESR at Week 156, and the analysis showed that the changes in plaque score were significantly lower in women than men (*p* = 0.0147) and in patients treated with ABT than in those treated with csDMARDs (*p* = 0.0303), whereas hypertension was identified as a risk factor for increased plaque score (*p* = 0.0188) (Table 3). We next analyzed the effects of use of antidiabetic agents, antihypertensive agents, statin, and steroids. These treatments did not significantly alter the change in plaque score (Additional file 2, Supplemental Table 31).

**Table 3:** Factors affecting the extent of changes in plaque score using a linear mixed regression model

	Applied with the disease activity, DAS-28-ESR, at baseline	Applied with the disease activity, DAS-28-ESR, at Week 156
	Plaque score Regression coefficient (95% confidence interval) *P* value	
Female sex	-1.12 (-2.106 – -0.134) 0.0264*	-1.221 (-2.197 – -0.246) 0.0147*
Age (≥ 65 years)	-0.33 (-1.033–0.373) 0.3536	-0.373 (-1.074–0.329) 0.2944
BMI (≥ 25 kg/m^2^)	-0.105 (-1.408–0.838) 0.8262	-0.059 (-0.166–0.048) 0.2767
Current or ex-smoker	-0.359 (-1.177–0.458) 0.3850	-0.335 (-1.144–0.475) 0.4138
Diabetes mellitus	-0.346 (-1.33–0.638) 0.4873	-0.49 (-1.467–0.487) 0.3219
Hypertension	0.84 (0.119–1.561) 0.0230*	0.86 (0.146–1.575) 0.0188*
Dyslipidemia	0.131 (-0.579–0.842) 0.7146	0.227 (-0.476–0.931) 0.5230
DAS28-ESR (≥ 3.2)	-0.203 (-1.037–0.631) 0.6303	0.263 (-0.546–1.072) 0.5202
ABT treatment	-0.711 (-1.462–0.04) 0.0632	-0.771 (-1.466 – -0.075) 0.0303*

Abbreviations: ABT: abatacept; BMI: body mass index; DAS28: disease activity score in 28 joints; ESR: erythrocyte sedimentation rate

### Secondary endpoints

#### EULAR response

**Fig. 1:**
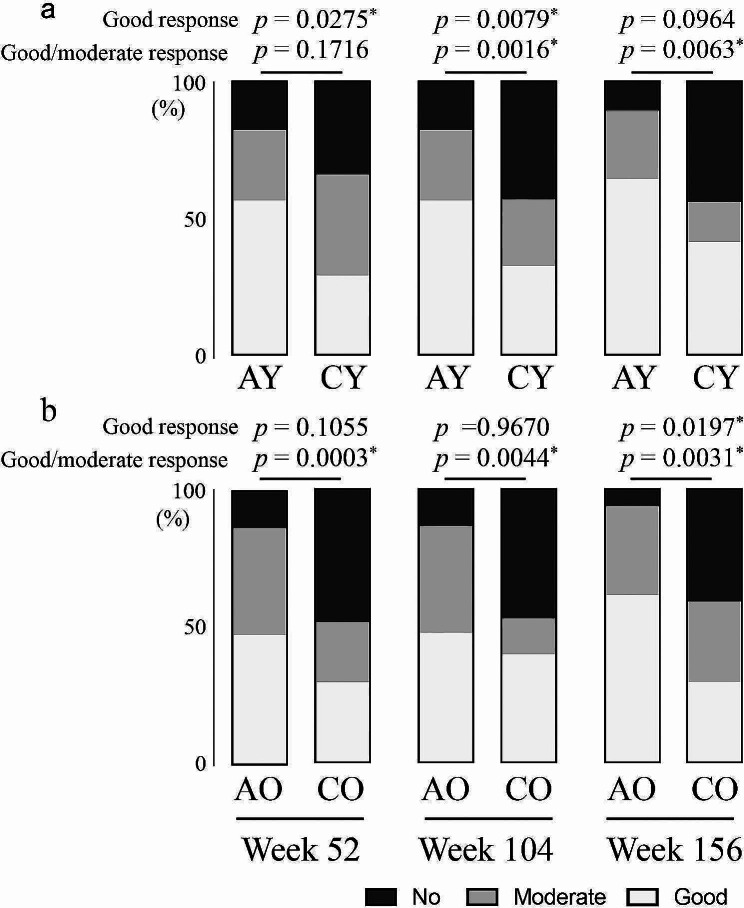
Proportions of RA patients with the indicated EULAR responses. **a** Comparison between AY and CY groups. **b** Comparison between AO and CO groups. *p < 0.05. AO: older patients taking abatacept; AY: younger patients taking abatacept; CO: older patients taking conventional synthetic disease-modifying antirheumatic drugs; CY: younger patients taking conventional synthetic disease-modifying antirheumatic drugs; RA: rheumatoid arthritis

Figure 1a and b show the proportions of patients with EULAR response at Weeks 52, 104, and 156. In the comparison between AY and CY groups, the proportions of patients with good response were significantly higher in the ABT group than in the csDMARD group at Weeks 52 and 104, but the difference did not reach statistical significance at Week 156 (Fig. 1a). The proportions of patients with good or moderate response were also significantly higher in the ABT group than in the csDMARD group at Weeks 104 and 156, but the difference did not reach statistical significance at Week 52. In comparing AO and CO groups, the proportions of patients with good response were higher in the ABT group at Week 156, but the difference did not reach statistical significance at Weeks 52 and 104 (Fig. 1b). The proportions of patients with good or moderate response were significantly higher in the ABT group at all time points. The proportions of patients with good response and good or moderate response were not significantly different between younger vs. older patients in ABT-treated groups (AY vs. AO).

#### Changes in DAS28-ESR, SDAI, CDAI, HAQ at Week 52, 104, and 156 in the ABT and csDMARD groups

Compared with patients treated with csDMARDs, decrease in RA disease activity measures, DAS28-ESR, SDAI, and CDAI; improvement in physical function assessment per HAQ were significantly greater in the ABT group at all time points in both younger and older patients (see Additional file 2, Supplemental Table 4). Decrease in DAS28-ESR, SDAI, CDAI, or HAQ were not significantly different between younger vs. older patients in ABT-treated groups (AY vs. AO).

The proportions of patients with a good response, good or moderate response, and changes in DAS28-ESR, SDAI, CDAI, and HAQ were significantly greater with ABT (AY + AO) than with csDMARDs (CY + CO) at all time points (see Additional file 2, Supplemental Table 5).

#### Baseline matching for subgroups AY and CY, and AO and CO

As the disease activity was higher in the ABT group at baseline, propensity-score matching was performed between AY and CY, and AO and CO using baseline patient characteristics. After matching, the baseline data showed that DAS28-ESR, SDAI, CDAI, and HAQ were no longer significantly different (see Additional file 2, Supplemental Table 6).

The post-matched cases were compared in the ABT and csDMARD groups at younger and older ages. The ABT group tended to have a greater proportion of good EULAR responses and good or moderate responses in older patients, and a greater range of changes in DAS28-ESR, SDAI, CDAI, and HAQ in both younger and older patients at Weeks 52 and 104 (see Additional file 2, Supplemental Table 7). At Week 156, significant differences were noted between AO and CO for proportions of patients with good and moderate EULAR response, and changes in DAS28-ESR, SDAI, and CDAI.

#### Baseline matching for AY and AO groups

Similarly, in the ABT-treated group, younger and older patients were matched. DAS28-ESR, SDAI, CDAI, and HAQ were not significantly different in the matched cases (see Additional file 2, Supplemental Table 8).

After matching, younger and older patients treated with ABT were compared. There were no significant differences regarding the proportion of good EULAR responses and good or moderate responses and in changes in DAS28-ESR, SDAI, CDAI, and HAQ at any time point (see Additional file 2, Supplemental Table 9).

#### Baseline matching ABT and csDMARD groups

Baseline data for the ABT and csDMARD groups were matched for younger and older patients combined. In the matched cases, DAS28-ESR, SDAI, CDAI, and HAQ were not significantly different (see Additional file 2, Supplemental Table 10).

After matching, the ABT group and the csDMARD group were compared. In the matched cases, the proportion of patients with good or moderate response, changes in DAS28-ESR, SDAI, CDAI, and HAQ was significantly greater in the ABT group than in the csDMARD group at Week 156 (see Additional file 2, Supplemental Table 11).

#### ABT retention rate and reasons for discontinuing ABT

**Fig. 2:**
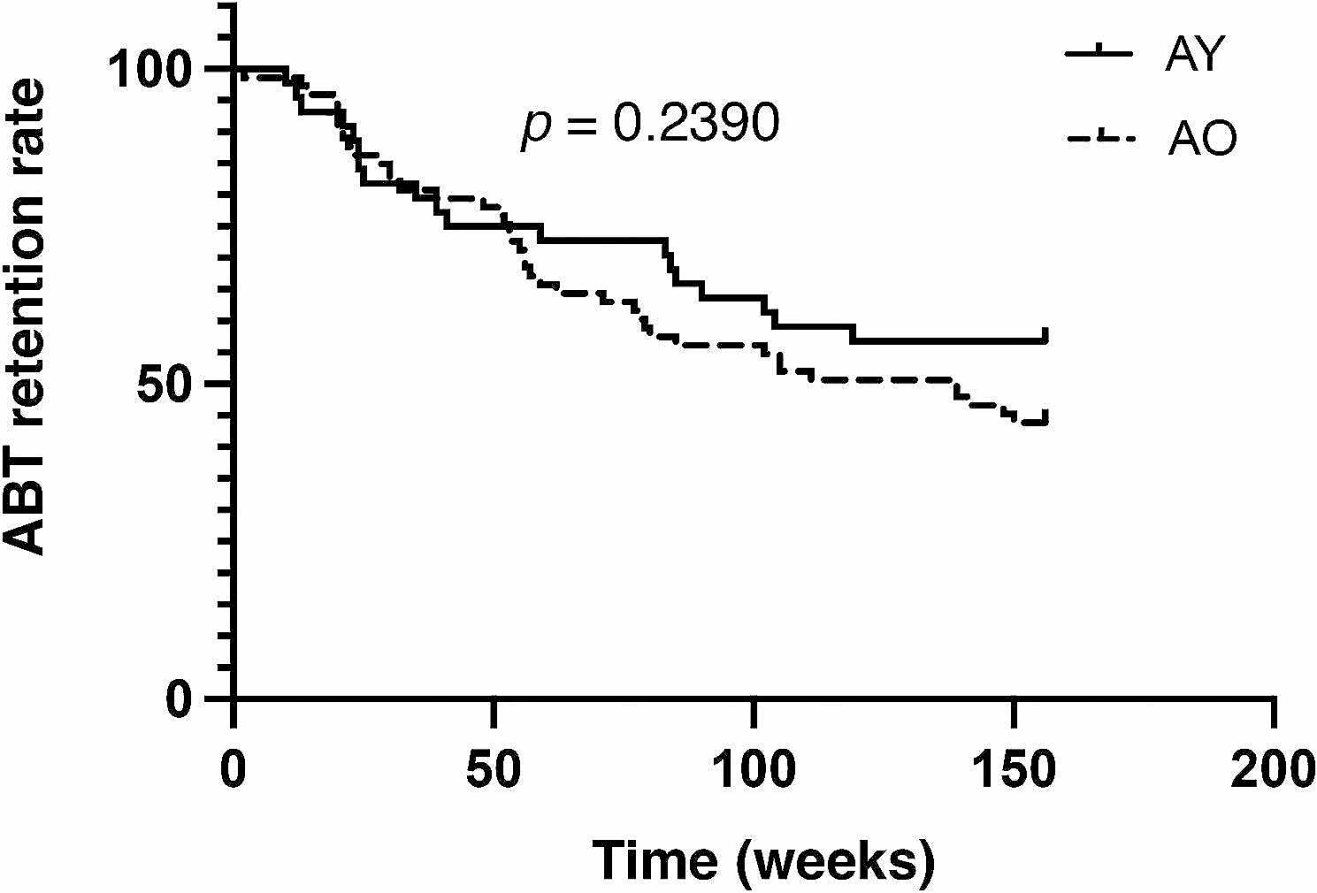
Kaplan–Meier curve for ABT retention rate. ABT: abatacept; AO: older patients taking abatacept; AY: younger patients taking abatacept

ABT retention rates were compared for younger and older patients. There was no significant difference between younger and older patients with RA (Fig. 2). In the AY group, two patients discontinued ABT because of AEs, six because of inadequate efficacy, and ten because of patient preference. In AO, 13 patients discontinued ABT because of AEs, 14 because of inadequate efficacy, and 9 because of patient preference (see Additional file 2, Supplemental Table 12) AEs up to Week 156.

#### AEs

There were significantly more AEs in the AY (50%) vs. CY (19.5%, *p* < 0.05) group. SAEs were significantly more common in older patients than in younger patients in both the ABT (27.4% vs. 11.5%, *p* < 0.05, respectively) and csDMARD groups (36.0% vs. 4.9%, *p* < 0.05, respectively). There were no significant differences in SAEs by type between the ABT and csDMARD groups. Among the SAEs, infections tended to be more common in the ABT group than in the csDMARD group, although this difference was not statistically significant (Table 4). Regarding deaths that occurred during the study, one patient died in the CO group due to a traffic accident. The cause of death was unrelated to the study treatment.

**Table 4:** Summary of adverse events over 156 weeks

	Abatacept		csDMARDs	
	AY	AO	CY	CO
Total number of adverse events	26 (50.0)^*^	46 (63.0)	8 (19.5)	30 (60.0)^$^
Serious adverse events	6 (11.5)	20 (27.4)^$^	2 (4.9)	18 (36.0)^$^
Infection	3 (5.8)	9 (12.3)		2 (4.0)
Pneumonia	1 (1.9)	4 (5.5)		2 (4.0)
Pulmonary Mycobacterium avium complex infection	2 (3.8)			1 (2.0)
Herpes zoster		2 (2.7)		
Cellulitis		1 (1.4)		
Septic arthritis		1 (1.4)		
Periodontal disease		1 (1.4)		
Malignancy	1 (1.9)	6 (8.2)		3 (6.0)
Lung cancer		2 (2.7)		1 (2.0)
Breast tumor		1 (1.4)		1 (2.0)
Lymphadenopathy		1 (1.4)		1 (2.0)
Prostate cancer		1 (1.4)		
Bladder cancer		1 (1.4)		
Subcutaneous tumor	1 (1.9)			
Others				
Bone fracture		3 (4.1)	2 (4.9)	
Joint surgery		2 (2.7)		3 (6.0)
Diabetes mellitus				2 (4.0)
Heart failure				2 (4.0)
Interstitial pneumonia		1 (1.4)		
Hyponatremia				1 (2.0)
Hypothyroidism		1 (1.4)		
Gallstones	1 (1.9)			
Retinal detachment				1 (2.0)
Hypersensitivity	1 (1.9)			
Subdural hematoma				1 (2.0)
Hydrocephalus				1 (2.0)
Death				1 (2.0) (due to traffic accident)

^*^p < 0.05, AY vs. CY. ^$^*p* < 0.05, AY vs. AO or CY vs. CO. Abbreviations: AO: older patients taking abatacept; AY: younger patients taking abatacept; CO: older patients taking conventional synthetic disease-modifying antirheumatic drugs; csDMARDs: conventional synthetic disease-modifying antirheumatic drugs; CY, younger patients taking conventional synthetic disease-modifying antirheumatic drugs

## Discussion

This study aimed to evaluate the effects of ABT on atherosclerosis and cardiovascular risk, at 3 years (156 weeks), comparing the results between treatment with ABT and csDMARDs in older and young groups and continue to evaluate the long-term efficacy and safety of ABT for patients with RA by age. Few prospective studies have evaluated RA treatment and outcomes in elderly patients, despite this being the predominantly affected population, and due to the chronicity of the disease, it is important to obtain long-term efficacy and safety data. Furthermore, this study is the first to report on the effect of ABT on atherosclerosis in patients with RA. Regarding the main findings for the primary outcome, mean IMT, max IMT, and plaque score increased less in the ABT group than in the csDMARD group, although differences were mostly not significantly different, but AO patients had a significantly lower increase in plaque score compared with CO patients. Multivariate analysis showed significantly lower increases in plaque score with ABT treatment, indicating that the progression of atherosclerosis was decelerated by treatment with ABT. The study also found that ABT had a long-term therapeutic effect in suppressing arthritis, with no significant difference in effect between older and younger patients. There were no significant differences in ABT retention rates between older and younger patients. SAEs, especially infection, tended to be more frequent in the ABT group, although no significant differences between groups were found.

Studies have previously reported that the incidence of cardiovascular disease was less in patients treated with ABT compared with those treated with TNF inhibitors [27, 28]. However, the effects of ABT on atherosclerosis observed in the present study are novel. Several studies have shown the usefulness of using ultrasonography to assess carotid plaques and determine plaque scores as a means of detecting early signs of atherosclerosis and predicting the risk of coronary heart disease and ischemic stroke [26, 29, 30]. Of note, although the risk of cardiovascular disease is higher in patients with higher disease activity than lower disease activity [31], we showed that ABT inhibited an increase in plaque score even when considering disease activity by multivariate analysis. While direct effects of ABT on arterial stiffness have yet to be reported, the ORACLE Arthritis Study [22] aims to elucidate the immunological and biological underpinnings of RA and atherosclerosis via detailed examinations of atherosclerosis progression and disease activity using high-sensitivity, high-throughput autoantibodies screening technologies. The results of the ORACLE Arthritis Study have yet to be published. It is expected that the present novel findings, along with the results of undergoing investigations, will further enable the understanding of RA and atherosclerosis, which will benefit the cardiovascular risk management and outcomes of these patients.

While the mechanism of abatacept on atherosclerosis inhibition is unclear, inflammatory cells play a role in the development of atherosclerosis [32]. In the atherosclerotic plaque, T cells produce various cytokines that may further contribute to plaque formation [33]. As ABT has been shown to inhibit T-cell activation, it could potentially reduce plaque scores [34]. Reportedly, plaques in unstable angina patients present clonally expanded peripheral blood CD4 T cells lacking CD28 expression (CD4^+^CD28^null^) [35], which are also increased in patients with RA [36]. CD4^+^CD28^null^ T cells contribute to monocyte activation in the plaque microenvironment by producing interferon-γ [37]. Patients with persistent CD4^+^CD28^null^ T-cell expansion show preclinical atherosclerotic changes [36]. However, ABT treatment decreased circulating CD4^+^CD28^null^ T cells [38]; thus, ABT may inhibit atherosclerosis by reducing these cells.

In this study, the long-term efficacy of ABT was confirmed at 156 weeks and no difference in efficacy was found between older and younger patients with RA. The present results confirm our findings on the efficacy of ABT vs. csDMARDs at weeks 12 and 24 observed in this population of older and younger patients with RA [23]. Furthermore, when comparing overall ABT and csDMARD groups, the proportions of patients with a good response, good or moderate response, and changes in DAS28-ESR, SDAI, CDAI, and HAQ were significantly greater with ABT at all time points, which is similar to the effectiveness results of a 3-year post-marketing surveillance of ABT for patients for RA in Japan. In that study, patients with a mean age of 63.1 years achieved significant decreases from baseline in DAS28-ESR and HAQ, among other effectiveness measures, with long-term ABT treatment [39]. A 5-year long-term study concluded similar improvements in measures, such as joint damage, disease activity, and physical function, among Japanese patients with RA [40].

The retention rate of ABT by 156 weeks did not differ by age. This finding is aligned with a recent study of the clinical effectiveness and long-term retention of ABT in elderly patients with RA using real-world data in which the retention rate did not vary across patient age groups (mean ages of 52.7, 67.7 and 78.1 years) and patient age was not a significant predictor of treatment discontinuations due to AEs [41].

Regarding safety, infections, as SAEs, were more common in the ABT group than in the csDMARD group, as well as among older vs. younger patients. There were only a few cases of infection, with the most common being pneumonia (AY, *n* = 1; AO, *n* = 4; CY, *n* = 0, and CO, *n* = 2), although no statistically significant differences were noted. These findings are similar to those in the previous report of this study [23] and previously published studies [12, 42, 43]. We should still exercise caution when using ABT, especially in the elderly, who may have a higher likelihood of developing infections.

The main limitations of this study were the observational design, relatively small sample size, limited number of cases after propensity-score matching, and limited generalizability owing to the exclusively Japanese population. In addition, only 118 patients in enrolled 216 patients completed the observation period of 156 weeks. The final number of patients was small and could alter the results of multivariate analysis.

## Conclusions

To conclude, ABT may decelerate atherosclerosis progression. The efficacy of ABT was maintained during the 3-year observation, without differences in older and younger patients. No new safety signals were detected, the retention rate remained high and there were no differences in older vs. younger patients. ABT may be useful for patients with high risk for cardiovascular disease, such as older patients.

### Electronic supplementary material

Below is the link to the electronic supplementary material.

Supplementary Material 1

Supplementary Material 2

## Data Availability

No datasets were generated or analysed during the current study.

## Abbreviations

ABT: Effect of abatacept
RA: Rheumatoid arthritis
AY: Younger patients taking abatacept
AO: Older patients taking abatacept
csDMARDS: Conventional synthetic disease-modifying antirheumatic drugs
CY: Younger patients taking conventional synthetic disease-modifying antirheumatic drugs
CO: Older patients taking conventional synthetic disease-modifying antirheumatic drugs
IMT: Intima-media thickness
b/tsDMARDS: Biologic and targeted synthetic disease-modifying antirheumatic drugs
ABT: Abatacept
TNF: Tumor necrosis factor
CD: Cluster of differentiation
AEs: Adverse events
EULAR: European League Against Rheumatism
PWV: Pulse wave velocity
DA28-ESR: disease activity score as measured across 28 joints using the erythrocyte sedimentation rate
SDAI: Simple Disease Activity Index
CDAI: Clinical Disease Activity Index
HAQ: Health assessment questionnaire (HAQ)
SAEs: Serious adverse events
