# Supplementary material for: Long-term effects of abatacept on atherosclerosis and arthritis in older vs. younger patients with rheumatoid arthritis: 3-year results of a prospective, multicenter, observational study
Source: Arthritis Res Ther. 2024 Apr 17;26:87. doi: 10.1186/s13075-024-03323-8 (PMC11022315; doi:10.1186/s13075-024-03323-8)
Supplement: Supplementary file 1 — Supplementary Material 1 [file 13075_2024_3323_MOESM1_ESM.docx]

**Additional file 1**

**Appendix.**

**List of collaborators**

ABT-ATS study group: Z. Yamada, S. Masuoka, S. Mizutani, S. Yamada, M. Kawazoe, H. Sato, M. Kaburaki, S. Muraoka, K. Kaneko, T. Nanki, Division of Rheumatology, Department of Internal Medicine, Toho University School of Medicine (Omori), Tokyo, Japan; W. Hirose, Hirose Clinic of Rheumatology, Saitama, Japan; Y. Kimura, K. Asako, H. Kikuchi, H. Kono, Department of Internal Medicine, Teikyo University School of Medicine, Tokyo, Japan; K. Nishimura, Department of Orthopaedic Surgery, Teikyo University School of Medicine, Tokyo, Japan; N. Abe, S. Tanimura, S. Yasuda, T. Atsumi, Department of Rheumatology, Endocrinology and Nephrology, Graduate School of Medicine and Faculty of Medicine, Hokkaido University, Hokkaido, Japan; Y. Komano, Department of Rheumatology, Jujo Takeda Rehabilitation Hospital, Kyoto, Japan; H. Kawano, J. Kishi, Y. Nishioka, Department of Respiratory Medicine and Rheumatology, Graduate School of Biomedical Sciences, Tokushima University, Tokushima, Japan; T Hidaka, Institute of Rheumatology, Zenjinkai Miyazaki-Zenjinkai Hospital, Miyazaki, Japan; S. Nakashima, Y. Takeuchi, H. Dobashi, Department of Internal Medicine, Division of Hematology, Rheumatology and Respiratory Medicine, Faculty of Medicine, Kagawa University, Kagawa, Japan; T Kasama, Division of Rheumatology, Department of Medicine, Showa University School of Medicine, Tokyo, Japan; D. Kanai, A. Ihata, Department of Rheumatology, Yokohama Minami Kyosai Hospital, Kanagawa, Japan; M. Inoo, Utazu Hospital, Ayauta-gun, Kagawa, Japan; K. Suemori, H. Hasegawa, Department of Hematology, Clinical Immunology and Infectious Diseases, Ehime University Graduate School of Medicine, Ehime, Japan; T. Okano, M. Tada, Department of Orthopaedic Surgery, Osaka City University Graduate School of Medicine, Osaka, Japan; S. Tsuboi, Department of Rheumatology, Shizuoka Kosei Hospital, Shizuoka, Japan; K. Kubo, T. Sugihara, Department of Medicine and Rheumatology, Tokyo Metropolitan Geriatric Hospital, Tokyo, Japan; S. Tsunoda, H. Sano, Division of Rheumatology, Department of Internal Medicine Hyogo College of Medicine, Hyogo, Japan; R. Yoshimi, Department of Stem Cell and Immune Regulation, Yokohama City University Graduate School of Medicine, Kanagawa, Japan; Y. Shimizu, S. Fukaya, T. Odani, Third Department of Internal Medicine, Obihiro-Kosei General Hospital, Hokkaido, Japan; K. Amano, Department of Immunology and Rheumatology, Saitama Medical Center, Saitama Medical University, Saitama, Japan; Y. Inoue, H. Kameda, Division of Rheumatology, Department of Internal Medicine, Toho University School of Medicine (Ohashi), Tokyo, Japan; T. Nagai, Department of Rheumatology and Infectious Diseases, Kitasato University School of Medicine, Kanagawa, Japan (Currently, T. Nagai, Department of Rheumatology, Minaminagano Medical Center Shinonoi General Hospital, Nagano, Japan) ; K. Ohmura, Department of Rheumatology and Clinical Immunology, Graduate School of Medicine, Kyoto University, Kyoto, Japan; S. Hirata, Department of Clinical Rheumatology, Kumamoto University Graduate School of Medicine, Kumamoto, Japan; K. Takagi, Department of Rheumatology, Sainokuni Higashi Omiya Medical Center, Saitama, Japan; Y. Inoue, K. Nakano, K. Saito, Y. Tanaka, The First Department of Internal Medicine, School of Medicine, University of Occupational and Environmental Health, Japan, Kitakyushu, Japan; Y. Takahashi, Yu-Family Clinic, Miyagi, Japan; Y. Okano, Department of Internal Medicine and Center for Arthritis and Rheumatic Disease, Kawasaki Municipal Kawasaki Hospital, Kanagawa, Japan; N. Hagino, Department of Rheumatology, Teikyo University Chiba Medical Center, Chiba, Japan; T. Sawada, Department of Rheumatology, Tokyo Medical University Hospital, Tokyo, Japan; T. Tsuru, PS Clinic, Fukuoka, Japan; H. Hagiyama, Department of Rheumatology, Yokohama City Minato Red Cross Hospital, Kanagawa, Japan.
