# Supplementary material for: Long-term effects of abatacept on atherosclerosis and arthritis in older vs. younger patients with rheumatoid arthritis: 3-year results of a prospective, multicenter, observational study
Source: Arthritis Res Ther. 2024 Apr 17;26:87. doi: 10.1186/s13075-024-03323-8 (PMC11022315; doi:10.1186/s13075-024-03323-8)
Supplement: Supplementary file 2 — Supplementary Material 2 [file 13075_2024_3323_MOESM2_ESM.docx]

**Additional file 2**

**Supplemental Figure 1. Patient disposition.**

**
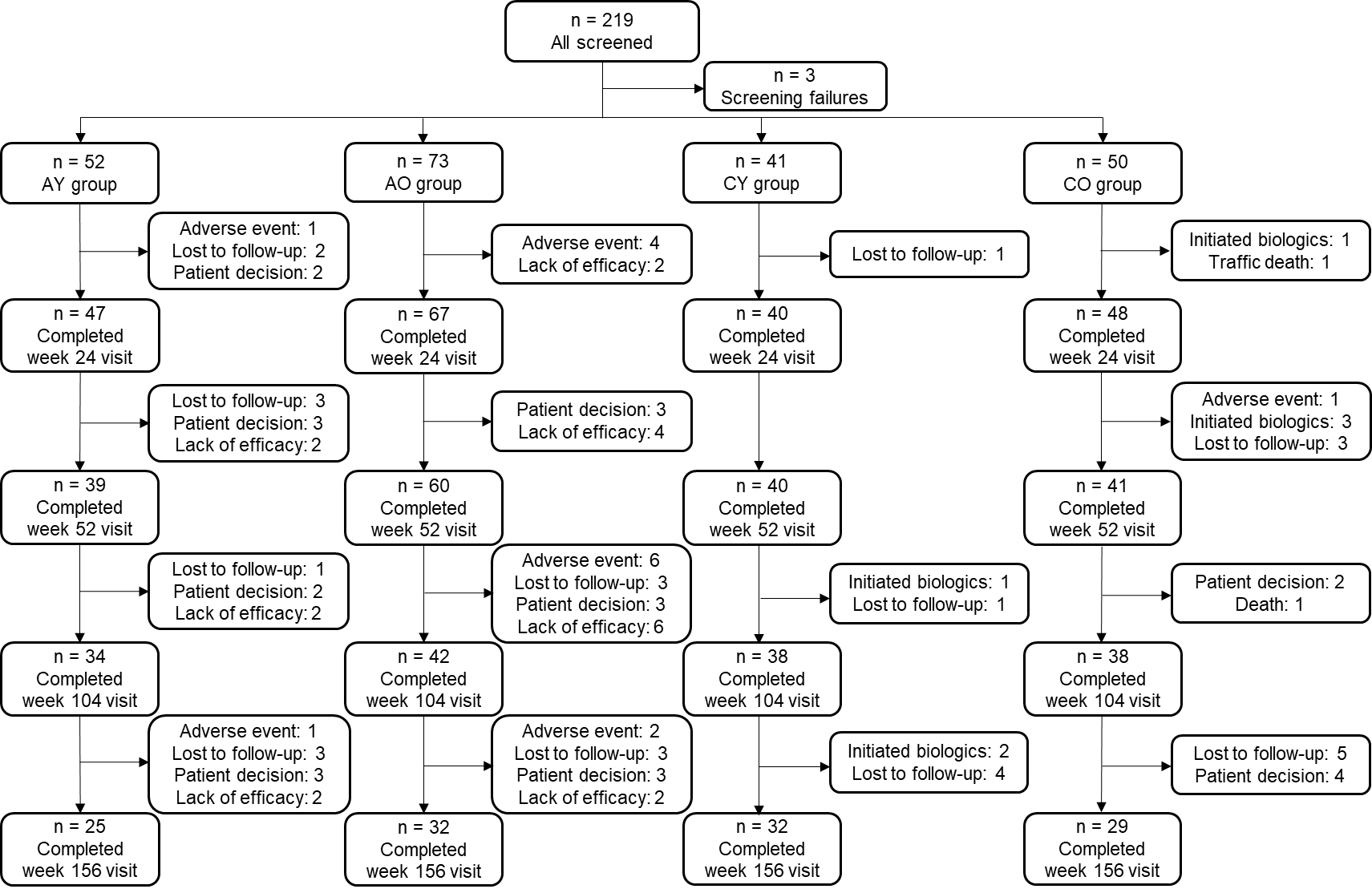
**

AO, older patients receiving abatacept; AY, younger patients receiving abatacept; CO, older patients receiving conventional synthetic disease-modifying antirheumatic drugs; CY, younger patients receiving conventional synthetic disease-modifying antirheumatic drugs.

**Supplemental Table 1**. Baseline characteristics of patients with rheumatoid arthritis in the overall population, according to treatment.

|  | Abatacept | csDMARDs | Total |
| --- | --- | --- | --- |
|  | Overall (n=125) | Overall (n=91) | (n=216) |
| Age (years) | 65.4 ± 14.2 | 64.2 ± 13.4 | 64.9 ± 13.8 |
| Female sex | 101 (80.8) | 79 (86.8) | 180 (83.3) |
| DAS28-ESR | 4.9 ± 1.2 | 3.5 ± 1.0^*^ | 4.3 ± 1.3 |
| SDAI | 23.4 ± 11.1 | 13.0 ± 6.8^*^ | 19.0 ± 10.8 |
| CDAI | 21.3 ± 10.0 | 12.4 ± 6.6^*^ | 17.6±9.8 |
| HAQ score | 0.9 ± 0.8 | 0.4 ± 0.7^*^ | 0.7 ± 0.8 |
| RF-positive | 107 (85.6) | 67 (73.6)^*^ | 174 (80.6) |
| ACPA-positive | 109 (87.2) | 69 (75.8) | 178 (82.4) |
| Steroid use | 58 (46.4) | 28 (30.8)^*^ | 86 (39.8) |
| Prednisolone (mg) | 5.8 ± 4.3 | 3.8 ± 1.4^*^ | 5.2 ± 3.8 |
| MTX use | 78 (62.4) | 51 (56.0) | 129 (59.7) |
| Additional treatment initiated during this study | |  |  |
| Abatacept | 125 (100) |  |  |
| MTX | - | 20 (22.0) | - |
| Salazosulfapyridine | - | 9 (9.8) | - |
| Bucillamine | - | 23 (25.3) | - |
| Tacrolimus | - | 7 (7.7) | - |
| Iguratimod | - | 29 (31.9) | - |
| Leflunomide | - | 3 (3.3) | - |
| Mean intima-media thickness (IMT) of common carotid artery (mm) | | | |
| Left | 0.753 ± 0.202 | 0.729 ± 0.156 | 0.743 ± 0.183 |
| Right | 0.739 ± 0.240 | 0.715 ± 0.147 | 0.728 ± 0.205 |
| Max intima-media thickness (IMT) of common carotid artery, bulbus, bifurcation and internal carotid artery (mm) | | | |
| Left | 0.929 ± 0.275 | 0.916 ± 0.235 | 0.923 ± 0.258 |
| Right | 0.922 ± 0.387 | 0.899 ± 0.217 | 0.912 ± 0.323 |
| Plaque score | 3.434 ± 2.708 | 3.233 ± 2.505 | 3.346 ± 2.615 |
| Pulse wave velocity (PWV) (cm/sec) | | | |
| Left | 1568.1 ± 441.2 | 1504.8 ± 355.7 | 1540.5 ± 406.4 |
| Right | 1549.2 ± 409.6 | 1507.7 ± 346.5 | 1531.0 ± 382.9 |

Results are shown as mean ± standard deviation or n (%). ^*^p<0.05, abatacept vs csDMARDs. ACPA, anti-cyclic citrullinated peptide antibody; CDAI, clinical disease activity index; csDMARDs, conventional synthetic disease-modifying antirheumatic drugs; DAS28, disease activity score in 28 joints; ESR, erythrocyte sedimentation rate; HAQ, Health Assessment Questionnaire; SDAI, simple disease activity index.

**Supplemental Table 2.** Intima-media thickness, plaque score, and pulse wave velocity changes at Weeks 52, 104, and 156.

|  | Week 52 | | | Week 104 | | | Week 156 | | |
| --- | --- | --- | --- | --- | --- | --- | --- | --- | --- |
| AY vs CY | | | | | | | | | |
|  | Abatacept | csDMARDs | *p-*value | Abatacept | csDMARDs | *p*-value | Abatacept | csDMARDs | *p*-value |
| Mean intima-media thickness of common carotid artery | | | | | | | | | |
| Left | 0.003 ± 0.118 | -0.003 ± 0.101 | 0.8205 | -0.003 ± 0.135 | 0.001 ± 0.080 | 0.8650 | 0.032 ± 0.116 | 0.030 ± 0.084 | 0.9420 |
| Right | 0.001 ± 0.116 | 0.009 ± 0.114 | 0.7486 | 0.024 ± 0.149 | 0.027 ± 0.099 | 0.9362 | 0.028 ± 0.095 | 0.061 ± 0.107 | 0.2088 |
| Max intima-media thickness of common carotid artery, bulbus, bifurcation and internal carotid artery | | | | | | | | | |
| Left | -0.011 ± 0.165 | -0.009 ± 0.132 | 0.9698 | -0.024 ± 0.172 | -0.011 ± 0.117 | 0.7435 | 0.007 ± 0.158 | 0.026 ± 0.113 | 0.6123 |
| Right | 0.014 ± 0.177 | -0.016 ± 0.142 | 0.4064 | 0.016 ± 0.205 | 0.017 ± 0.127 | 0.9842 | 0.033 ± 0.120 | 0.049 ± 0.137 | 0.6204 |
| Plaque score | 0.215 ± 2.016 | -0.120 ± 1.947 | 0.4919 | -0.174 ± 1.696 | -0.291 ± 1.742 | 0.8024 | -0.059 ± 1.343 | 0.051 ± 1.686 | 0.7942 |
| Pulse wave velocity | | | | | | | | | |
| Left | 64.2 ± 227.8 | -15.7 ± 110.1 | 0.0537 | 29.7 ± 150.3 | 26.9 ± 87.2 | 0.9268 | 58.3 ± 173.7 | 37.6 ± 137.1 | 0.6140 |
| Right | 22.6 ± 189.9 | -24.5 ± 124.1 | 0.1986 | 28.7 ± 160.0 | 16.0 ± 109.6 | 0.7075 | 74.2 ± 191.5 | 19.3 ± 143.6 | 0.2195 |
| AO vs CO | | | | | | | | | |
|  | Abatacept | csDMARDs | P value | Abatacept | csDMARDs | P value | Abatacept | csDMARDs | P value |
| Mean intima-media thickness of common carotid artery | | | | | | | | | |
| Left | 0.024 ± 0.126 | 0.020 ± 0.120 | 0.8706 | -0.002 ± 0.126 | 0.017 ± 0.124 | 0.5060 | 0.013 ± 0.115 | 0.033 ± 0.145 | 0.5622 |
| Right | 0.046 ± 0.147 | 0.024 ± 0.106 | 0.4018 | -0.005 ± 0.146 | 0.025 ± 0.120 | 0.3299 | 0.012 ± 0.146 | 0.004 ± 0.140 | 0.8348 |
| Max intima-media thickness (IMT) of common carotid artery, bulbus, bifurcation, and internal carotid artery | | | | | | | | | |
| Left | 0.071 ± 0.227 | 0.041 ± 0.190 | 0.4695 | 0.017 ± 0.173 | 0.016 ± 0.264 | 0.9983 | 0.008 ± 0.144 | 0.014 ± 0.290 | 0.9134 |
| Right | 0.089 ± 0.268 | 0.038 ± 0.319 | 0.4037 | 0.009 ± 0.232 | 0.017 ± 0.239 | 0.8797 | -0.023 ± 0.259 | -0.017 ± 0.254 | 0.9227 |
| Plaque score | 0.304 ± 2.056 | 0.268 ± 2.073 | 0.9378 | -0.390 ± 1.711 | 0.333 ± 2.302 | 0.1488 | -0.803 ± 1.865 | 0.397 ± 2.130 | **0.0302**^*^ |
| Pulse wave velocity | | | | | | | | | |
| Left | 8.4 ± 275.8 | -52.9 ± 217.0 | 0.2222 | -34.5 ±498.5 | -10.3 ±189.0 | 0.7766 | 22.6 ± 500.7 | 111.6 ± 498.5 | 0.4786 |
| Right | 32.0 ± 293.2 | -36.0 ± 273.6 | 0.2451 | 7.1 ± 321.9 | -24.4 ±218.2 | 0.6210 | 47.8 ± 281.6 | 46.4 ± 243.8 | 0.9841 |
| Abatacept (AY+AO) vs csDMARDs (CY+CO); t-test was used at each time point. | | | | | | | | | |
|  | Abatacept | csDMARDs | P value | Abatacept | csDMARDs | P value |  |  |  |
| Mean intima-media thickness of common carotid artery | | | | | | | | | |
| Left | 0.016 ± 0.123 | 0.009 ± 0.111 | 0.7055 | -0.003 ± 0.129 | 0.009 ± 0.104 | 0.5444 |  |  |  |
| Right | 0.028 ± 0.136 | 0.017 ± 0.109 | 0.5623 | 0.008 ± 0.147 | 0.026 ± 0.109 | 0.4033 |  |  |  |
| Max intima-media thickness of common carotid artery, bulbus, bifurcation, and internal carotid artery | | | | | | | | | |
| Left | 0.039 ± 0.207 | 0.016 ± 0.165 | 0.4305 | 0.000 ± 0.172 | 0.003 ± 0.204 | 0.9224 |  |  |  |
| Right | 0.059 ± 0.238 | 0.011 ± 0.249 | 0.1985 | 0.012 ± 0.219 | 0.017 ± 0.191 | 0.8844 |  |  |  |
| Plaque score | 0.266 ± 2.028 | 0.091 ± 2.011 | 0.5934 | -0.296 ± 1.694 | 0.046 ± 2.071 | 0.3177 |  |  |  |
| Pulse wave velocity | | | | | | | | | |
| Left | 30.8 ± 257.8 | -34.5 ± 172.6 | **0.0465**^*^ | -6.0 ± 383.9 | 8.6 ± 146.7 | 0.7645 |  |  |  |
| Right | 28.2 ± 255.2 | -30.3 ± 212.0 | 0.0992 | 16.9 ± 260.2 | -3.9 ± 171.9 | 0.5762 |  |  |  |

Results are reported as change of the measured value at the time point compared with the baseline value. ^*^P values < 0.05 are highlighted in bold. Abbreviations: AO: older patients taking abatacept; AY: younger patients taking abatacept; CO: older patients taking conventional synthetic disease-modifying antirheumatic drugs; csDMARDs: conventional synthetic disease-modifying antirheumatic drugs; CY: younger patients taking conventional synthetic disease-modifying antirheumatic drugs. Comparison between Abatacept (AY+AO) vs csDMARDs (CY+CO) at week 156 is shown in Table 2.

**Supplemental Table 3.** Factors affecting the extent of changes in plaque score using a linear mixed regression model.

|  | Applied with the disease activity, DAS-28-ESR, at baseline | Applied with the disease activity, DAS-28-ESR, at Week 156 |
| --- | --- | --- |
|  | Plaque score  Regression coefficient (95% confidence interval)  P value | |
| Female sex | -1.229 (-2.284 – -0.174)  **0.0229*** | -1.304 (-2.346 – -0.262)  **0.0147*** |
| Age (≥65 years) | -0.197 (-0.953 – 0.56)  0.6074 | -0.222 (-0.983 – 0.539)  0.5636 |
| BMI (≥25 kg/m^2^) | -0.201 (-1.172 – 0.77)  0.6816 | -0.277 (-1.247 – 0.692)  0.5714 |
| Current or ex-smoker | -0.408 (-1.252 – 0.435)  0.3389 | -0.442 (-1.283 – 0.398)  0.2989 |
| Antidiabetic agents | 0.283 (-0.904 – 1.471)  0.6369 | 0.137 (-1.035 – 1.309)  0.8171 |
| Antihypertensive agents | -0.019 (-0.825 – 0.787)  0.9630 | -0.032 (-0.844 – 0.779)  0.9372 |
| Statin | 0.302 (-0.588 – 1.192)  0.5022 | 0.341 (-0.554 – 1.236)  0.4513 |
| Steroids | 0.213 (-0.519 – 0.945)  0.5647 | 0.207 (-0.527 – 0.94)  0.5774 |
| DAS28-ESR (≥3.2) | -0.353 (-1.198 – 0.492)  0.4084 | 0.211 (-0.627 – 1.049)  0.6183 |
| ABT treatment | -0.625 (-1.396 – 0.146)  0.1107 | -0.733 (-1.461 – -0.004)  **0.0488*** |

Abbreviations: ABT: abatacept; BMI: body mass index; DAS28: disease activity score in 28 joints; ESR: erythrocyte sedimentation rate.

**Supplemental Table 4**. Detailed efficacy results of abatacept and csDMARDs in younger and older patients with rheumatoid arthritis.

|  | Younger |  |  | Older |  |  |
| --- | --- | --- | --- | --- | --- | --- |
|  | Abatacept (AY) | csDMARDs (CY) | P value | Abatacept (AO) | csDMARDs (CO) | P value |
| Week 52 |  |  |  |  |  |  |
| Change in DAS28-ESR from baseline | -2.017 ± 1.365 | -0.929 ± 0.893 | **0.0001** | -2.110 ± 1.232 | -0.630 ± 1.124 | **<0.0001** |
| Change in SDAI from baseline | -15.708 ± 10.691 | -5.895 ± 5.746 | **<0.0001** | -16.135 ± 11.408 | -5.252 ± 5.566 | **<0.0001** |
| Change in CDAI from baseline | -14.331 ± 9.847 | -6.055 ± 5.883 | **<0.0001** | -14.765 ± 10.943 | -5.120 ± 5.354 | **<0.0001** |
| Change in HAQ from baseline | -0.372 ± 0.515 | -0.072 ± 0.243 | **0.0017** | -0.322 ± 0.576 | -0.091 ± 0.361 | **0.0158** |
| Week 104 |  |  |  |  |  |  |
| Change in DAS28-ESR from baseline | -2.319 ± 1.227 | -0.829 ± 1.013 | **<0.0001** | -2.168 ± 1.304 | -0.877 ± 1.119 | **<0.0001** |
| Change in SDAI from baseline | -17.987 ± 9.601 | -5.866 ± 6.480 | **<0.0001** | -17.779 ± 12.389 | -5.822 ± 6.018 | **<0.0001** |
| Change in CDAI from baseline | -16.335 ± 8.540 | -5.859 ± 6.030 | **<0.0001** | -16.291 ± 11.475 | -6.089 ± 6.105 | **<0.0001** |
| Chang in HAQ from baseline | -0.463 ± 0.470 | -0.069 ± 0.205 | **<0.0001** | -0.418 ± 0.593 | -0.092 ± 0.373 | **0.0045** |
| Week 156 |  |  |  |  |  |  |
| Change in DAS28-ESR from baseline | -2.208 ± 1.345 | -0.893 ± 1.197 | **0.0002** | -2.496 ± 1.394 | -0.822 ± 1.109 | **<0.0001** |
| Change in SDAI from baseline | -19.209 ± 10.553 | -6.498 ± 6.763 | **<0.0001** | -18.376 ± 12.666 | -5.784 ± 6.481 | **<0.0001** |
| Change in CDAI from baseline | -17.218 ± 9.465 | -6.349 ± 6.440 | **<0.0001** | -16.808 ± 11.941 | -5.488 ± 6.156 | **<0.0001** |
| Chang in HAQ from baseline | -0.438 ± 0.490 | -0.046 ± 0.256 | **0.0005** | -0.434 ± 0.586 | -0.048 ± 0.793 | **0.0054** |

Results are shown as mean ± standard. P values < 0.05 are highlighted in bold. AO, older patients receiving abatacept; AY, younger patients receiving abatacept; CDAI, clinical disease activity index; CO, older patients receiving conventional synthetic disease-modifying antirheumatic drugs; csDMARDs, conventional synthetic disease-modifying antirheumatic drugs; CY, younger patients receiving conventional synthetic disease-modifying antirheumatic drugs; DAS28, disease activity score in 28 joints; ESR, erythrocyte sedimentation rate; HAQ, Health Assessment Questionnaire; SDAI, simple disease activity index.

**Supplemental Table 5**. Detailed efficacy results for the abatacept and csDMARD overall groups.

|  | Abatacept | csDMARDs | P value |
| --- | --- | --- | --- |
| Week 52 |  |  |  |
| Proportion of patients with good EULAR response (%) | 51.0 | 29.1 | **0.0053** |
| Proportion of patients with good or moderate EULAR response (%) | 84.7 | 58.2 | **0.0002** |
| Change in DAS28-ESR from baseline | -2.073 ± 1.281 | -0.774 ± 1.024 | **<0.0001** |
| Change in SDAI from baseline | -15.965 ± 11.074 | -5.566 ± 5.628 | **<0.0001** |
| Change in CDAI from baseline | -14.592 ± 10.471 | -5.581 ± 5.606 | **<0.0001** |
| Chang in HAQ from baseline | -0.342 ± 0.550 | -0.082 ± 0.307 | **0.0001** |
| Week 104 |  |  |  |
| Proportion of patients with good EULAR response (%) | 53.5 | 36.0 | **0.0497** |
| Proportion of patients with good or moderate EULAR response (%) | 88.7 | 54.7 | **<0.0001** |
| Change in DAS28-ESR from baseline | -2.234 ± 1.264 | -0.853 ± 1.061 | **<0.0001** |
| Change in SDAI from baseline | -17.870 ± 11.182 | -5.843 ± 6.208 | **<0.0001** |
| Change in CDAI from baseline | -16.311 ± 10.228 | -5.976 ± 6.028 | **<0.0001** |
| Chang in HAQ from baseline | -0.438 ± 0.538 | -0.081 ± 0.299 | **<0.0001** |
| Week 156 |  |  |  |
| Proportion of patients with good EULAR response (%) | 62.7 | 34.8 | **0.0029** |
| Proportion of patients with good or moderate EULAR response (%) | 91.5 | 56.5 | **<0.0001** |
| Change in DAS28-ESR from baseline | -2.359 ± 1.367 | -0.858 ± 1.147 | **<0.0001** |
| Change in SDAI from baseline | -18.778 ± 11.600 | -6.146 ± 6.587 | **<0.0001** |
| Change in CDAI from baseline | -17.003 ± 10.747 | -5.925 ± 6.270 | **<0.0001** |
| Change in HAQ from baseline | -0.435 ± 0.539 | -0.047 ± 0.388 | **<0.0001** |

Results are shown as mean ± standard deviation unless otherwise stated. P values < 0.05 are highlighted in bold. CDAI, clinical disease activity index; csDMARDs, conventional synthetic disease-modifying antirheumatic drugs; DAS28, disease activity score in 28 joints; ESR, erythrocyte sedimentation rate; EULAR, European League Against Rheumatism; HAQ, Health Assessment Questionnaire; SDAI, simple disease activity index.

**Supplemental Table 6.** Baseline characteristics of patients with rheumatoid arthritis after propensity-score matching to compare AY and CY, and AO and CO.

|  | AY; n=25 | CY; n=25 | P value | AO; n=31 | CO; n=31 | P value |
| --- | --- | --- | --- | --- | --- | --- |
| Age (years) | 54.3 ± 9.6 | 51.7 ± 8.8 | 0.3222 | 74.4 ± 6.1 | 74.5 ± 5.8 | 0.9157 |
| Female sex | 22 (88.0) | 22 (88.0) | 1.0000 | 24 (77.4) | 27 (87.1) | 0.5061 |
| DAS28-ESR | 3.9 ± 0.7 | 3.7±0.9 | 0.4125 | 4.4 ± 1.2 | 4.2 ± 1.1 | 0.4708 |
| SDAI | 14.6 ± 5.0 | 14.4 ± 5.8 | 0.8933 | 17.8 ± 9.6 | 15.6 ± 8.3 | 0.3249 |
| CDAI | 14.2 ± 5.0 | 14.0 ± 5.7 | 0.8980 | 16.9 ± 9.3 | 14.7 ± 8.1 | 0.3105 |
| HAQ score | 0.5 ± 0.6 | 0.3 ± 0.5 | 0.2167 | 0.8 ± 0.8 | 0.8 ± 0.9 | 0.8422 |

Results are shown as mean ± standard deviation or n (%).AO, older patients receiving abatacept; AY, younger patients receiving abatacept; CDAI, clinical disease activity index; CO, older patients receiving conventional synthetic disease-modifying antirheumatic drugs; CY, younger patients receiving conventional synthetic disease-modifying antirheumatic drugs; DAS28, disease activity score in 28 joints; ESR, erythrocyte sedimentation rate; HAQ, Health Assessment Questionnaire; SDAI, simple disease activity index.

**Supplemental Table 7**. Detailed efficacy results of abatacept and csDMARDs in younger and older patients with rheumatoid arthritis after propensity-score matching.

|  | Younger |  |  | Older |  |  |
| --- | --- | --- | --- | --- | --- | --- |
|  | Abatacept (AY) | csDMARDs (CY) | P value | Abatacept (AO) | csDMARDs (CO) | P value |
| Week 52 |  |  |  |  |  |  |
| Proportion of patients with good EULAR response (%) | 46.7 | 44.4 | 1.0000 | 44.4 | 30.0 | 0.8607 |
| Proportion of patients with good or moderate EULAR response (%) | 66.7 | 72.2 | 1.0000 | 88.9 | 60.0 | 0.3649 |
| Change in DAS28-ESR from baseline | -1.272 ± 1.040 | -1.125 ± 0.999 | 0.6835 | -1.784 ± 0.836 | -0.725 ± 1.107 | **0.0305**^*^ |
| Change in SDAI from baseline | -9.042 ± 6.963 | -7.928 ± 6.399 | 0.6350 | -10.398 ± 7.007 | -7.047 ± 4.992 | 0.2539 |
| Change in CDAI from baseline | -8.840 ± 6.687 | -8.265 ± 6.415 | 0.7996 | -9.521 ± 6.609 | -6.460 ± 4.766 | 0.2701 |
| Chang in HAQ from baseline | -0.083 ± 0.495 | -0.144 ± 0.312 | 0.6821 | -0.319 ± 0.656 | -0.087 ± 0.306 | 0.3528 |
| Week 104 |  |  |  |  |  |  |
| Proportion of patients with good EULAR response (%) | 71.4 | 45.5 | 0.2360 | 50.0 | 43.4 | 0.9036 |
| Proportion of patients with good or moderate EULAR response (%) | 92.9 | 72.7 | 0.2911 | 75.0 | 60.9 | 0.5092 |
| Change in DAS28-ESR from baseline | -1.655 ± 0.741 | -1.175 ± 0.997 | 0.1082 | -1.696 ± 1.454 | -1.000 ± 1.104 | 0.0892 |
| Change in SDAI from baseline | -10.850 ± 4.191 | -8.117 ± 7.233 | 0.1608 | -11.018 ± 11.182 | -6.710 ± 6.386 | 0.1390 |
| Change in CDAI from baseline | -10.429 ± 4.415 | -7.973 ± 6.727 | 0.1949 | -10.808 ± 10.460 | -7.070 ± 6.585 | 0.1779 |
| Chang in HAQ from baseline | -0.358 ± 0.543 | -0.103 ± 0.249 | 0.1050 | -0.394 ± 0.668 | -0.114 ± 0.457 | 0.1240 |
| Week 156 |  |  |  |  |  |  |
| Proportion of patients with good EULAR response (%) | 50.0 | 52.3 | 1.0000 | 70.6 | 26.3 | **0.0202**^*^ |
| Proportion of patients with good or moderate EULAR response (%) | 83.3 | 66.7 | 0.5301 | 94.1 | 62.8 | **0.0340**^*^ |
| Change in DAS28-ESR from baseline | -1.299 ± 0.824 | -1.243 ± 1.225 | 0.8773 | -2.176 ± 1.523 | -0.833 ± 1.608 | **0.0052**^*^ |
| Change in SDAI from baseline | -10.660 ± 5.287 | -8.666 ± 7.607 | 0.3938 | -13.108 ± 10.502 | -5.906 ± 7.625 | **0.0269**^*^ |
| Change in CDAI from baseline | -10.136 ± 5.218 | -8.395 ± 7.216 | 0.4410 | -12.381 ± 9.947 | -5.342 ± 7.199 | **0.0199**^*^ |
| Chang in HAQ from baseline | -0.385 ± 0.597 | -0.060 ± 0.305 | 0.0990 | -0.514 ± 0.637 | -0.132 ± 0.613 | 0.0717 |

Results are shown as mean ± standard. ^*^P values < 0.05 are highlighted in bold. AO, older patients receiving abatacept; AY, younger patients receiving abatacept; CDAI, clinical disease activity index; CO, older patients receiving conventional synthetic disease-modifying antirheumatic drugs; csDMARDs, conventional synthetic disease-modifying antirheumatic drugs; CY, younger patients receiving conventional synthetic disease-modifying antirheumatic drugs; DAS28, disease activity score in 28 joints; ESR, erythrocyte sedimentation rate; EULAR, European League Against Rheumatism; HAQ, Health Assessment Questionnaire; SDAI, simple disease activity index.

**Supplemental Table 8**. Baseline characteristics of AO and AY groups adjusted after propensity-score matching.

|  | AY; n=38 | AO; n=38 | P value |
| --- | --- | --- | --- |
| Age (years) | 52.2 ± 10.4 | 74.2 ± 5.7 | **<0.0001** |
| Female sex | 29 (76.3) | 26 (68.4) | 0.6079 |
| DAS28-ESR | 4.7 ± 1.1 | 4.6 ± 1.3 | 0.7080 |
| SDAI | 22.0 ± 10.2 | 21.0 ± 11.5 | 0.7074 |
| CDAI | 20.2 ± 9.0 | 19.3 ± 10.7 | 0.6796 |
| HAQ score | 0.7 ± 0.6 | 0.7 ± 0.5 | 0.9801 |

Results are shown as mean ± standard deviation or n (%). P values < 0.05 are highlighted in bold. AO, older patients receiving abatacept; AY, younger patients receiving abatacept; CDAI, clinical disease activity index; DAS28, disease activity score in 28 joints; ESR, erythrocyte sedimentation rate; HAQ, Health Assessment Questionnaire; SDAI, Simple Disease Activity Index.

**Supplemental Table 9**. Detailed efficacy results of younger and older patients treated with abatacept after propensity-score matching.

|  | AY | AO | P value |
| --- | --- | --- | --- |
| Week 52 |  |  |  |
| Proportion of patients with good EULAR response (%) | 60.0 | 59.3 | 1.0000 |
| Proportion of patients with good or moderate EULAR response (%) | 84.0 | 88.9 | 0.9128 |
| Change in DAS28-ESR from baseline | -1.964 ± 1.381 | -2.162 ± 1.193 | 0.5830 |
| Change in SDAI from baseline | -15.262 ± 10.959 | -15.827 ± 11.339 | 0.8558 |
| Change in CDAI from baseline | -13.864 ± 10.290 | -14.318 ± 10.610 | 0.8761 |
| Chang in HAQ from baseline | -0.280 ± 0.484 | -0.306 ± 0.458 | 0.8461 |
| Week 104 |  |  |  |
| Proportion of patients with good EULAR response (%) | 64.0 | 45.8 | 0.3218 |
| Proportion of patients with good or moderate EULAR response (%) | 96.0 | 79.2 | 0.1735 |
| Change in DAS28-ESR from baseline | -2.356 ± 1.265 | -1.924 ± 1.464 | 0.2752 |
| Change in SDAI from baseline | -18.176 ± 9.964 | -14.583 ± 13.754 | 0.3026 |
| Change in CDAI from baseline | -16.416 ± 8.924 | -13.775 ± 12.524 | 0.4022 |
| Chang in HAQ from baseline | -0.504 ± 0.494 | -0.350 ± 0.443 | 0.2356 |
| Week 156 |  |  |  |
| Proportion of patients with good EULAR response (%) | 54.5 | 70.0 | 0.4765 |
| Proportion of patients with good or moderate EULAR response (%) | 86.4 | 95.0 | 0.6701 |
| Change in DAS28-ESR from baseline | -2.073 ± 1.426 | -2.446 ± 1.471 | 0.4101 |
| Change in SDAI from baseline | -18.726 ± 11.171 | -17.578 ± 13.595 | 0.7661 |
| Change in CDAI from baseline | -16.630 ± 9.971 | -15.914 ± 12.676 | 0.8370 |
| Change in HAQ from baseline | -0.460 ± 0.524 | -0.399 ± 0.439 | 0.6787 |

Results are shown as mean ± standard deviation unless otherwise stated. AO, older patients receiving abatacept; AY, younger patients receiving abatacept; CDAI, clinical disease activity index; DAS28, disease activity score in 28 joints; ESR, erythrocyte sedimentation rate; EULAR, European League Against Rheumatism; HAQ, Health Assessment Questionnaire; SDAI, simple disease activity index.

**Supplemental Table 10**. Baseline characteristics of patients with rheumatoid arthritis in the overall population after propensity-score matching to compare abatacept and csDMARDs.

|  | Abatacept  Overall (n=58) | csDMARDs  Overall (n=58) | P value |
| --- | --- | --- | --- |
| Age (years) | 63.5 ± 12.7 | 65.7 ± 13.2 | 0.3618 |
| Female sex | 49 (84.5) | 51 (87.9) | 0.7877 |
| DAS28-ESR | 4.1 ± 0.9 | 3.9 ± 1.1 | 0.3528 |
| SDAI | 15.6 ± 5.9 | 15.0 ± 7.1 | 0.6406 |
| CDAI | 14.8 ± 6.0 | 14.3 ± 6.9 | 0.6699 |
| HAQ score | 0.6 ± 0.6 | 0.6 ± 0.8 | 0.8444 |

Results are shown as mean ± standard deviation or n (%). CDAI, clinical disease activity index; DAS28, disease activity score in 28 joints; ESR, erythrocyte sedimentation rate; HAQ, Health Assessment Questionnaire; SDAI, simple disease activity index.

**Supplemental Table 11**. Detailed efficacy results for the abatacept and csDMARDs overall groups after propensity-score matching.

|  | Abatacept | csDMARDs | P value |
| --- | --- | --- | --- |
| Week 52 |  |  |  |
| Proportion of patients with good EULAR response (%) | 54.5 | 35.3 | 0.1807 |
| Proportion of patients with good or moderate EULAR response (%) | 81.8 | 61.8 | 0.1212 |
| Change in DAS28-ESR from baseline | -1.620 ± 0.920 | -0.989 ± 0.924 | **0.0068**^*^ |
| Change in SDAI from baseline | -10.689 ± 6.372 | -7.598 ± 5.450 | **0.0310**^*^ |
| Change in CDAI from baseline | -10.572 ± 6.862 | -7.503 ± 5.584 | **0.0470**^*^ |
| Chang in HAQ from baseline | -0.197 ± 0.484 | -0.132 ± 0.250 | 0.4925 |
| Week 104 |  |  |  |
| Proportion of patients with good EULAR response (%) | 54.3 | 43.8 | 0.4677 |
| Proportion of patients with good or moderate EULAR response (%) | 80.0 | 62.5 | 0.1405 |
| Change in DAS28-ESR from baseline | -1.557 ± 1.112 | -1.052 ± 1.065 | 0.0410 |
| Change in SDAI from baseline | -9.791 ± 6.969 | -7.389 ± 6.376 | 0.1127 |
| Change in CDAI from baseline | -9.574 ± 6.620 | -7.406 ± 6.337 | 0.1380 |
| Chang in HAQ from baseline | -0.330 ± 0.509 | -0.148 ± 0.338 | 0.0676 |
| Week 156 |  |  |  |
| Proportion of patients with good EULAR response (%) | 62.1 | 37.8 | 0.0711 |
| Proportion of patients with good or moderate EULAR response (%) | 90.0 | 60.0 | **0.0125**^*^ |
| Change in DAS28-ESR from baseline | -1.757 ± 1.239 | -0.921 ± 1.233 | **0.0062**^*^ |
| Change in SDAI from baseline | -11.187 ± 7.051 | -7.175 ± 7.298 | **0.0231**^*^ |
| Change in CDAI from baseline | -10.638 ± 6.828 | -6.833 ± 6.925 | **0.0233**^*^ |
| Chang in HAQ from baseline | -0.438 ± 0.518 | -0.106 ± 0.441 | **0.0056**^*^ |

Results are shown as mean ± standard deviation unless otherwise stated. ^*^P values < 0.05 are highlighted in bold. CDAI, clinical disease activity index; csDMARDs, conventional synthetic disease-modifying antirheumatic drugs; DAS28, disease activity score in 28 joints; ESR, erythrocyte sedimentation rate; EULAR, European League Against Rheumatism; HAQ, Health Assessment Questionnaire; SDAI, simple disease activity index.

**Supplemental Table 12**. Reason for discontinuation of abatacept.

|  | Younger |  |  | Older |  |  |
| --- | --- | --- | --- | --- | --- | --- |
| Week | 0-52 | 52-104 | 104-156 | 0-52 | 52-104 | 104-156 |
| Due to adverse events | |  |  |  |  |  |
| Rash | - | - | - | 1 | 1 | - |
| Infectious pneumonia | - | - | 1 | 2 | 2 | 1 |
| Lung cancer | - | - | - | - | 2 | - |
| Bladder cancer | - | - | - | 1 | - | - |
| Periodontal disease | - | - | - | - | 1 | - |
| Cellulitis | - | - | - | - | 1 | - |
| Hypersensitivity | 1 | - | - | - | - | - |
| Lack of efficacy | 2 | 2 | 2 | 6 | 6 | 2 |
| Patient decision | 5 | 2 | 3 | 3 | 3 | 3 |
